# Distribution of Integrons and Phylogenetic Groups among Enteropathogenic *Escherichia coli* Isolates from Children <5 Years of Age in Delhi, India

**DOI:** 10.3389/fmicb.2017.00561

**Published:** 2017-04-10

**Authors:** Taru Singh, Shukla Das, V. G. Ramachandran, Sayim Wani, Dheeraj Shah, Khan A. Maroof, Aditi Sharma

**Affiliations:** ^1^Microbiology, University College of Medical Sciences and Guru Teg Bahadur HospitalNew Delhi, India; ^2^Dermatology, University College of Medical Sciences and Guru Teg Bahadur Hospital, Dilshad GardenNew Delhi, India; ^3^Department of Minimal Access and Bariatric Surgery, Fortis Flt. Rajan Dhall HospitalNew Delhi, India; ^4^Pediatrics, University College of Medical Sciences and Guru Teg Bahadur HospitalNew Delhi, India; ^5^Community Medicine, University College of Medical Sciences, and Guru Teg Bahadur Hospital, Dilshad GardenNew Delhi, India; ^6^Vardhman Mahavir Medical College and Sardarjung HospitalNew Delhi, India

**Keywords:** enteropathogenic *Escherichia coli*, phylogenetic groups, multidrug resistance, integrons, gene cassettes

## Abstract

Integrons by means of horizontal gene transfer carry multidrug resistance genes (MDR) among bacteria, including *E. coli*. The aim of this study was to determine the antibiotic resistance profiles and the genes associated with them, to gain insights in the distribution of phylogroups, prevalence, and characterization of class 1, 2 and 3 integrons among Enteropathogenic *E. coli* (EPEC) isolates, from children upto 5 years of age from Delhi and National Capital Region (NCR), India. A total of 120 *E. coli* isolates, including 80 from diarrheagenic *E. coli* (cases) and 40 from healthy isolates (controls) were recruited in this study. After isolation of *E. coli*, screening for EPEC was done by conventional multiplex PCR. Antibiotic suseptibility test was performed using disk diffusion method and further confirmed by minimum inhibitory concentration (MICs) by *E*-test. The presence and characterization of integrons and antimicrobial resistance genes were performed by PCR and DNA sequencing. Phylogeny determination was carried out by quadruplex PCR. EPEC strains were found in 64 of the 80 diarrheagenic cases, out of which 38 were MDR. In the 40 healthy controls, 23 were found to be EPEC strain, out of which only 2 were MDR. Amongst 80 diarrheagenic cases, class 1 integron were observed in 43 isolates, class 2 integron in 12 isolates and 9 isolates were found with co-existence of both. Similarly, in healthy controls; class 1 integron in 9 and class 2 integron in 7 isolates were observed with co-existence in 3 isolates. None of the isolates included class 3 integron. The *dfr* was the most commonly identified gene cassette within the integron-positive isolates. Phylogenetic studies showed considerable representation of phylogroup B2 in both diarrheagenic cases and healthy controls. This study reiterates the importance of class 1 integron predominantly for acquisition of antibiotic resistance genes among EPEC isolates. Furthermore, it also ascertains the possible association between multidrug resistance and presence of integrons. Approximately 91% of isolates were easily assigned to their respective phylogroups. Assessment of the relationship between antibiotic resistance and dominant phylogroups detected was also attempted. This study also highlights the increased burden of antimicrobial resistance in healthy controls.

## Introduction

Childhood diarrhea is a predominant cause of child mortality worldwide, with an estimated of 2.2 million children getting affected in the developing countries alone (Ramana and Tamanna, 2012). Among the six known types of *E. coli*, Enteropathogenic *Escherichia coli* (EPEC) are one of the major causative agents of infantile diarrhea in developing countries. EPEC is classified into two subgroups; atypical EPEC (aEPEC) and typical EPEC (tEPEC) based on bundle-forming pili (*bfp*), present in typical *E. coli* (Trabulsi et al., 2002). EPEC adherence factor plasmid (pEAF) encodes bundle-forming pili which is essential for EPEC virulence, antigenicity, auto-aggregation, and localized adherence to epithelial cells (Trabulsi et al., 2002). The *bfpA* gene present on EAF plasmid, promotes the stabilization of bacteria by interconnecting within microcolonies. Intimin is a 94-kDa outer membrane protein encoded by *eae* gene, which is responsible for the adherence between bacteria and enterocyte membranes (Nataro and Kaper, 1998). The genetic determinants that produce A/E lesions are located on the locus of enterocyte effacement (LEE), a pathogenicity island that contains the genes *eae* encoding intimin (McDaniel et al., 1995). Attaching and effacing (A/E) lesions are the distinctive feature of EPEC infection, which are characterized by intimate attachment of the bacterium to the intestinal epithelial surface, effacement of surface-absorptive microvilli, and reorganization of actin filaments in the intestinal cell just beneath the site of attachment, which leads to the formation of pedestal-like structures (Moon et al., 1983; Croxen and Finlay, 2010). Atypical EPEC strains (lacking pEAF) are considered less virulent, as the genes encoding virulence factors are not only located on transmissible plasmids but also on transposons or bacteriophages (Trabulsi et al., 2002); nevertheless, these have not been proven to be less pathogenic (Levine et al., 1985).

The World Health Organization (WHO) has identified growing antibiotic resistance as a major problem among pediatric population. Many antibiotic resistance genes are located on plasmids, transposons, and integrons and they are greatly enhanced when they form part of a mobile gene cassette, since this provides for horizontal transfer by several mechanisms (White et al., 2001). The dihydrofolate reductase gene, *dfr*, plays a key role in maintaining intracellular folate homeostasis, cell growth and proliferation and is an important target for cytostatic drugs (Holger et al., 2011). Integrons commonly found in bacteria are involved in antibiotic resistance as they carry resistance genes in the form of gene cassettes. Integron-positive isolates have a greater tendency to acquire antibiotic resistance than isolates without integrons (White et al., 2001). The integrons are not mobile themselves with some exceptions (Hall and Collis, 1995) but are transported by transposons/plasmids (Barlow and Hall, 2002; Normark and Normark, 2002). There are mainly five classes of integrons known so far, each with a difference in sequence of integrase gene (Mazel, 2006). Integron carries antibiotic-resistance genes and is highly disseminated because of its close association with transposons, often embedded in conjugative plasmids (Cambray et al., 2010). Class 1 integrons were found to be more prevalent as compared to other classes of integrons (Phongpaichit et al., 2008; Rezaee et al., 2011). Most integrons carry dihydrofolate reductase gene (*dfr*), which confers resistance to trimethoprim and an aminoglycoside adenyltransferase gene (*aadA*), which confers resistance to streptomycin and spectinomycin (El-Najjar et al., 2010).

Extended spectrum β-lactamases (ESBL), AmpC β-lactamases, and carbapenemases extensively accrued by *Enterobacteriaceae* have now emerged as important nosocomial pathogens in India and other parts of the world (Dallenne et al., 2010; Jia et al., 2014). The ESBLs belonging to Group 2be (Bush et al., 1995) and AmpC beta-lactamases are well defined enzymes with wide range substrate specificity which could be chromosomal as well as plasmid mediated (Barlow and Hall, 2002; Babic et al., 2006). Carbapenems being stable in presence of ESBLs and AmpC enzymes are often the last choice of antibiotics in the treatment of infections due to multidrug-resistant *Enterobacteriaceae* isolates. Class 1 integrons contain bacterial resistance determinants to aminoglycosides as aided by aminoglycoside adenyltransferase (*aadA*), aminoglycoside acetyltransferases (*AACs*), and aminoglycoside phosphotransferases (*APHs*), which inactivate aminoglycosides by various identified mechanisms. The *sul1* gene that encodes resistance to sulphonamides, is found exclusively on large conjugative plasmids and on class 1 integron at the 3′ end (Hall and Collis, 1995).

Based on the presence of certain genes or DNA fragments, *E. coli* populations are categorized into eight major phylogenetic groups namely; A, B1, B2, C, D, E, F (belonging to *E. coli sensu stricto)* and clade I (belonging to *Escherichia* clade) which can be identified using a new method of Clermont et al. (2013). These genes have been identified as *chuA*, encoding for heme transport protein (Torres and Payne, 1997; Bonacorsi et al., 2000); *yjaA*, for a hypothetical protein with unknown function (Anton et al., 2015); *TSPE4.C2* as a putative lipase esterase-encoding gene (Clermont et al., 2013) with *arpA*, as an internal control for phylo-group F. Most of the commensal *E. coli* strains are mainly assigned to phylogroups A and B1 (Dobrindt, 2005; Kaper, 2005). However, for diarrheagenic *E. coli* (DEC), the arrangement of phylogroups is still unclear with multiple studies reporting diverse occurrences (Mosquito et al., 2015). Similarly, there is not much information available between the association of antibiotic resistance and phylogeny (Hannah et al., 2009).

The aim of this study was to determine antimicrobial resistance profiles and the genes associated with them, the prevalence and diversity of integrons, to identify the predominant phylogenetic groups, to analyze the possible relationship between presence of integrons and multidrug resistance in enteropathogenic *E. coli* isolates from diarrheagenic cases and healthy controls in children under 5 years of age in Delhi, India.

## Materials and methods

### Study design and ethical committee approval

During the study period (July 2013–July 2014), 80 stool samples were collected from children suffering from acute diarrhea (<72 h duration) from the Out-Patient Department (OPD) of a tertiary care hospital and 40 stool samples were also collected from healthy children (neither receiving antibiotics nor suffering from any disease). This study was carried out in accordance with the recommendations of “WHO guidelines, Institutional Ethics Committee—Human Research (IEC-HR) University College of Medical Sciences, University of Delhi, New Delhi” with written informed consent from all subjects. All subjects gave written informed consent in accordance with the Declaration of Helsinki. The study was approved by the “Institutional Ethics Committee—Human Research (IEC-HR) University College of Medical Sciences, University of Delhi, New Delhi.”

### Sample collection and processing

Fresh stool samples were collected in clean, leak-proof, labeled, sterile, and wide-mouthed plastic containers and were transported immediately to the laboratory and cultured. Up to five dark pink colonies (lactose fermentation) with the typical appearance of *E. coli* on MacConkey agar were selected and subjected to conventional biochemical tests for identifying *E. coli* such as gram staining (gram-negative and rod shaped bacterium), catalase test (+), oxidase test (−), glucose fermentation with production of gas, fermentation of other sugars (lactose, sucrose, maltose, and mannitol), nitrate reduction (+, reduces nitrate into nitrite), urease (−), Methyl red + Voges Proskauer (MR + and VP −), OF glucose test (glucose Fermenter), decarboxylase test [lysine (+), arginine (−) and ornithine (+/−)], indol test (+), Simon's citrate (−) and hydrogen sulfide (−) (Collee et al., 1996; Koneman et al., 1997). PCR for 16SrRNA gene was also performed, which was used as an internal quality control for *E. coli* confirmation (Wang et al., 2002).

### DNA extraction

‘Eluted lactose fermenting colonies on MacConkey agar were selected for DNA extraction using the commercial kit from Real Biotech Corporation, Taiwan as per manufacturer's guidelines. The extracted DNA was isolated in 100 μL of elution buffer. This DNA was used to perform conventional PCR for identification of genes associated with EPEC virulence, antibiotic resistance, class 1, 2, and 3 integrons, gene cassettes and different phylogenetic groups. Primers used for amplifying the various sequences were selected from previously published literature (Hollingshead and Vapnek, 1985; Yu and Kaper, 1992; Van de Klundert and Vliegenthart, 1993; Franke et al., 1994; Gunzburg et al., 1995; Levesque et al., 1995; Guardabassi et al., 2000; Mazel et al., 2000; Navia et al., 2003; Machado et al., 2005; Clermont et al., 2008, 2013; Kim et al., 2009; Dallenne et al., 2010; Naas et al., 2011; Manoharan et al., 2012; Lescat et al., 2013; Li et al., 2014; Table 1, Supplementary File).

**Table 1:** **Age and sex distribution of isolates in diarrheagenic cases and healthy controls**.

**Groups**	**Diarrheagenic cases (*****n*** = **80)**			**Healthy controls (*****n*** = **40)**			***P*-value**
**Age in years**	**M^#^**	**F^#^**	**EPEC (%) / Total isolates (%)**	**M^#^**	**F^#^**	**EPEC (%) / Total isolates (%)**	
(0–1)	20	11	31(38.75) / 35 (43.75)	10	1	11(27.5) / 18 (45)	0.03^*^
(1–3)	9	7	16(20) / 20 (25)	5	1	6(15) / 8 (20)	0.77
(3–5)	9	8	17(21.25) / 25 (31.25)	4	2	6(15) / 14 (35)	0.12
Total	38	26	64(80) / 80 (100)	19	4	23(57.5) / 40 (100)	0.00^*^

*Gene frequencies are presented as absolute numbers with percentage in parentheses*.

*EPEC, Enteropathogenic E. coli*.

* *significant p-value*.

# M, Male;

# *F, Female*.

### Detection of EPEC virulence

The criteria to determine typical enteropathogenic *E. coli* and atypical enteropathogenic *E. coli* were defined as follows: the presence of *eae* and *bfpA* genes for typical EPEC and presence of *eae* gene only depicts atypical *E. coli* (Kaper, 1996; Trabulsi et al., 2002). Typical EPEC strains (tEPEC) also contain the EPEC adherence factor (EAF) plasmid which carries genes encoding bundle-forming pili (BFP) (Baldini et al., 1983; Giron et al., 1991).

### Conventional multiplex PCR for detection of genes for EPEC virulence, antibiotic resistance, class 1, 2, and 3 integrons, gene cassettes, and phylogroups

For each set of multiplex PCR assay, 0.2 ml tubes were used, containing total volume of 25 μl of PCR mixture including, 2.5 μl buffer (10X), 1 μl dNTP's (200 uM), 1 μl MgCL2 (1.5 mM), 1 μL of each forward and reverse primers (10 μM), 5 μl of the extracted DNA and nuclease free water to make up the volume. All PCR reagents were purchased from Genei, Bangalore and amplifications were performed on an Eppendorf thermo cycler (Vidal et al., 2004). PCR amplification involved an initial denaturation step at 94°C for 10 min which was followed by 35 amplification cycles of 40 s at 94°C and 30 s at annealing temperature (as shown in Table 1, Supplementary File) and 40 s at 72°C and a final extension of 7 min at 72°C followed by a hold step at 4°C. Negative controls were PCR mixtures containing water in place of template DNA and for EPEC it was *E. coli* ATCC 11775, which is devoid of all the virulence genes (Janisiewicz et al., 1999). Amplified PCR products were stored at −20°C and analyzed by electrophoresis on 1.5% agarose gel (stained with ethidium bromide) at 125 volts with 15 mA current in an 18-slot apparatus for 30 min. A molecular marker of 100 bp (Fermentas) was used to determine the size of the amplicons (Sambrook et al., 1989). Uniplex PCR was also performed initially for confirmation of multiple genes.

### Antibiotic susceptibility testing

For initial screening, antimicrobial susceptibility testing was performed with 16 antimicrobial agents (HiMedia Laboratories Mumbai, India) namely; norfloxacin (10 μg), cefotaxime (30 μg), imipenem (10 μg), meropenem (10 μg), ceftazidime (30 μg), aztreonam (30 μg), nalidixic acid (30 μg), amoxicillin (20/10 μg), gentamicin (10 μg), ciprofloxacin (5 μg), ampicillin (10 μg), amikacin (30 μg), polymixin B (300 units/disc), cefotaxime + clavulanic acid (30/10 μg), ceftriaxone (30 μg) and Piperacillin+tazobactam (100/10 μg) on Mueller-Hinton agar plates by the Kirby Bauer disc diffusion method as per CLSI guidelines (CLSI, 2012) and later minimum inhibitory concentration (MIC) by E-strips (HiMedia Laboratories Mumbai, India) was performed for confirmation. Multidrug resistant (MDR) isolates show resistance to ≥3 antibiotic classes (Magiorakos et al., 2012). *Escherichia coli* (ATCC) strain 25922 was included as a quality control.

### Amplification and sequencing of gene cassette regions

Gene cassettes *aadA1/aadA2, dfrB1, dfrB2, dfrB3*, and *hep 58* and *hep 59* which bind to 3′ and 5′CS region of class 1 integrons were amplified using specific primers (Table 1, Supplementary File). Sequencing of the class I integrons gene cassettes (amplified by the primers *hep58* and *hep59*) were performed commercially (Yaazh xenomics, Chennai, India). In order to increase the accuracy of the results, sequencing was performed with both forward and reverse primers and sequences were compared against GenBank database by using Basic Local Alignment Search Tool (BLAST) and submitted in the NCBI database.

### Detection of antibiotic resistance genes

Antibiotic resistance genes of the beta-lactamase class namely; extended spectrum beta lactamases (ESBL); *TEM* (Temoneira), *SHV* (sulfhydryl variable), *CTX* (cefotaxime hydrolyzing capabilities), *OXA* (oxacillin hydrolyzing capabilities), Metallo beta-lactamases (MBL); *NDM-1* (New Delhi metallo beta lactamase), *IMP* (imipenem), *VIM* (Verona integron-encoded metallo-β-lactamase) and AmpC β-Lactamases (AmpC); *ACT* (AmpC type), *CMY* (cephamycins), *DHA* (Dhahran Hospital) and other antibiotic resistance genes namely; *sul-1* (sulphonamides), *tet-A* (tetracycline) and *aacC-1* (gentamicin) were determined by PCR (Table 1, Supplementary File).

### Molecular typing of isolates

All the isolates were screened for phylogenetic groups A, B1, B2, C, D, E, F, and Clade I using quadruplex multiplex PCR as described by Clermont et al. (2013). The *chuA* gene was present in all strains belonging to groups B2 and D and absent from all strains belonging to groups A and B1 which separates groups B2 and D from groups A and B1. The *yjaA* gene allows discrimination between group B2 and group D.

### Statistical analysis

Collected Data was statistically analyzed using Statistical Package for the Social Science (SPSS; Version 20.0). Chi square test and Fisher's exact test were used to determine the statistical significance of data. *P* < 0.05 was considered significant.

## Results

**Figure 1:**
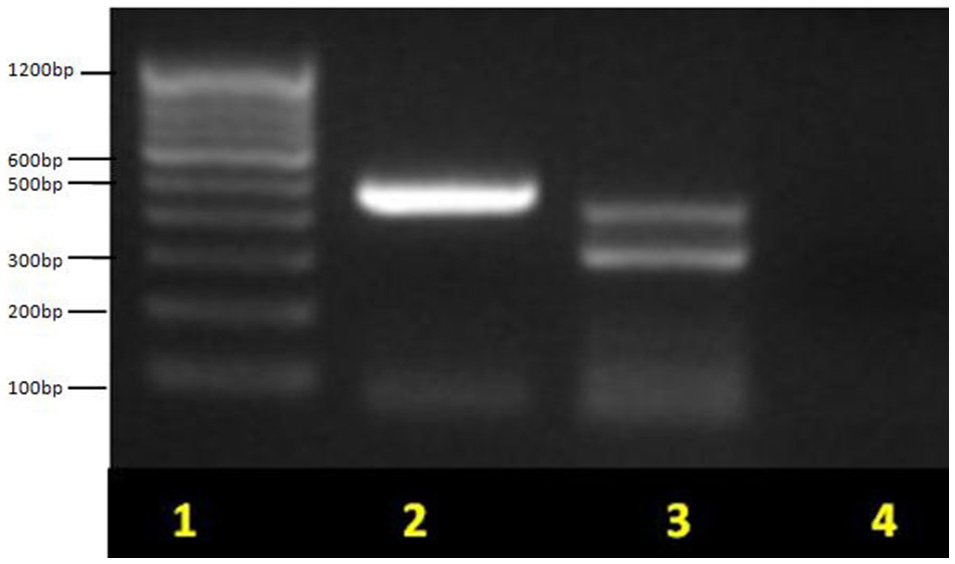
**Multiplex PCR for EPEC virulence genes showing typical EPEC and atypical EPEC on 1.5% agarose gel**. Lane 1, molecular weight marker (100 bp, Fermentas); lane 2, *eae* (482 bp) for atypical EPEC; lane 3, *bfpA* (326 bp) + *eae* (482 bp) for typical EPEC and lane 4, no template control (negative) control. Non-pathogenic *E. coli* ATCC 11775 was used as a negative control.

This study highlighted male prevalence in all the three age groups in diarrheagenic cases and healthy controls as shown in Table 1. The highest number of EPEC was found in the children with age up to 1 year (*P* < 0.05) while in the remaining two age groups, the percentage of EPEC isolates were almost similar in diarrheagenic cases (20–21.5%) and healthy controls (15% each), though not statistically significant as depicted in Table 1. The amplified PCR products of EPEC virulence for *eae, bfpA* and *eaf* genes on 1.5% agarose gel represents the uniplex PCR (Figure 1, Supplementary File), while Figure 1 shows that of multiplex PCR for all the three genes. Typical EPEC [*eae*+*bfpA(eaf)*] was found in 18.75% and 2.5% isolates (*P* = 0.001), atypical EPEC (*eae*) was found in 25% and 37.5% isolates (*P* = 0.673) while the total EPEC was found in 80% and 57.5% isolates (*P* = 0.012) of diarrheagenic cases and healthy controls respectively. 50/80 (62.5%) diarrheagenic cases (38 EPEC and 12 non-EPEC isolates) and 5/40 (12.5%) healthy controls (2 EPEC and 3 non-EPEC isolates) were multi drug resistant. The *bfpA* gene (usually encoded on the EAF plasmid) alone was found in 20% diarrheagenic cases and 17.5% healthy controls (*P* = 0.523). Three isolates were found to be positive for *eaf* (plasmid) gene in the absence of its virulence (*bfpA*) gene.

**Figure 2:**
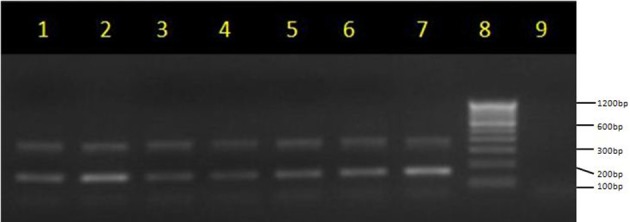
**Multiplex PCR for integrase genes on 1.5% agarose gel**. Multiplex PCR products of class 1 integrase (170 bp) and class 2 integrase genes (403 bp) lane 1–7: lane 8, molecular weight marker (100 bp, Fermentas); lanes 9, no template control (negative) control.

The amplified PCR products of integrase genes on 1.5% agarose gel represents the uniplex PCR for class 1 integron (160 bp) and class 2 integron (403 bp) (Figure 2, Supplementary Files). Figure 2 depicts the multiplex PCR for class 1 and class 2 integrase genes when compared with a 100 bp ladder. The distribution of integrons among EPEC and non-EPEC isolates are shown in Table 2, class 1 integron was found to be significantly (*p* = 0.018) associated with EPEC virulence, compared to class 2 integron, between diarrheagenic cases and healthy controls. Class 3 integron was not found in any of the isolates. No significant association was found when class 1 and 2 integrons were present together. The total number of integron-positive isolates in diarrheagenic cases and healthy controls among EPEC and non-EPEC isolates were also found to be highly significant (*p* = 0.003 and 0.011 respectively) as shown in Table 2. The occurrence of class 1 integron were found to be 3.14 and 2.295 times higher in EPEC and non-EPEC isolates compared to class 2 integron or both classes of integrons together (Odds ratio). However; in non-EPEC isolates a remarkable difference was found between integron carrying isolates in diarrheagenic cases and healthy controls. The occurrence of integrons was found to be 4.54 times higher (Odds ratio) in total EPEC isolates as compared to total non-EPEC isolates which were significantly different (*p* = 0.03) as indicated in Table 2.

**Table 2:** **Distribution of integron among EPEC and non-EPEC isolates**.

	**EPEC**				**Non-EPEC**				**EPEC vs. Non-EPEC**			
**Target gene**	**Cases (*n* = 64)**	**Controls (*n* = 23)**	***P*-value**	**Odds ratio (lower-upper)**	**Cases (*n* = 16)**	**Controls (*n* = 17)**	***P*-value**	**Odds ratio (lower-upper)**	**Total EPEC (*n* = 87)**	**Total non-EPEC (*n* = 33)**	***P*-value**	**Odds ratio (lower-upper)**
Integron 1	43 (67.18)	9 (39.13)	0.018^*^	3.14 (1.16–8.75)	9 (56.25)	6 (35.29)	0.227	2.295 (0.475–12.03)	52 (59.77)	15 (45.45)	0.158	1.774 (0.786–4.047)
Integron 2	12 (18.75)	7 (30.43)	0.245	0.699 (0.24–2.01)	4 (25)	3 (17.64)	0.636	1.535 (0.265–9.725)	19 (21.83)	7 (21.21)	0.940	1.037 (0.394–2.931)
Integron 1+ Integron 2	9 (14.06)	3 (13.04)	0.937	0.689 (0.15–3.01)	3 (18.75)	2 (11.76)	0.618	1.702 (0.221–16.21)	12 (13.79)	5 (15.15)	0.833	0.896 (0.293–3.059)
Total	64 (100)	19 (82.6)	0.003^*^	N.A	16 (100)	11 (64.7)	0.011^*^	N.A	83 (95.4)	27 (81.81)	0.03^*^	4.54 (1.159–19.48)

* *significant P-value*.

*Gene frequencies are presented as absolute numbers with percentage in parentheses*.

*EPEC, Enteropathogenic E. coli*.

Antibiotic susceptibility testing was performed for 16 antibiotic agents as mentioned above. MIC-test was also performed for selected antibiotics by E-strip method. For resistant isolates, MIC of norfloxacin was in the range of 16–256 μg/ml, amikacin (64–256 μg/ml), cefotaxime (4–256 μg/ml), Piperacillin/tazobactam (128–256 μg/ml), gentamicin (16–256 μg/ml) and ciprofloxacin (4–256 μg/ml). Table 3 shows the relation between antibiotic resistance and presence of integrons. In our study, resistance to gentamicin, cefotaxime, ceftazidime, nalidixic acid and aztreonam was found to be significantly associated with integrons. (*P* < 0.05) as shown in Table 3. Table 4 depicts the relationship between the presence of integron and occurrence of multidrug resistance. A strong association was found between MDR and presence of integrons (*P* = 0.01).

**Table 3:** **Association between antimicrobial resistance and integrons**.

**Antibiotic**	**Total resistant (120) No. (%)**	**Resistant isolates with int. genes (110) No. (%)**	**Association of resistance with integrion (*P*-value)**
Norfloxacin	26 (13)	14 (12.7)	0.07
Cefotaxime	67 (55.8)	31 (28.1)	0.000^*^
Imipenem	18 (15)	13 (11.8)	0.48
Meropenem	3 (2.5)	2 (1.8)	0.75
Ceftazidime	12 (10)	2 (1.8)	0.009^*^
Azetronam	11 (9.1)	1 (0.9)	0.004^*^
Nalidixic acid	14(11.6)	3 (2.7)	0.009^*^
Amoxicillin	3 (2.5)	1 (0.9)	0.41
Gentamicin	31 (25.8)	17 (15.4)	0.05^*^
Ciprofloxacin	12(10)	7 (6.3)	0.33
Ampicillin	30 (25)	26 (23.6)	0.53
Amikacin	23 (19.1)	14 (12.7)	0.19
Polymixin B	1 (0.8)	0 (0)	>0.99
Cefotaxime+ clavulanic acid	1 (0.8)	1 (0.9)	0.95
Ceftriaxone	2(1.6)	0 (0)	0.271
Piperacillin+tazobactam	21 (17.5)	12 (10.9)	0.16

* *significant p-value*.

*Frequencies are presented as absolute numbers with percentage in parentheses*.

**Table 4:** **Relation between presence of integron and MDR**.

**Integron**	**Antibiotic**		***P*-value**
	**MDR No. (%)**	**Other isolates No. (%)**	
Integron positive (*n* = 110)	54(98.18)	56 (86.15)	<0.0^*^
Integron negative (*n* = 10)	1(1.81)	9(13.84)	
Total	55	65	

* *significant p-value*.

*Frequencies are presented as absolute numbers with percentage in parentheses*.

MDR, multi drug resistance

### Integron and gene cassettes in *E. coli* isolates

PCR and sequencing analysis of class I integrons gene cassettes (*hep58* and *hep59*) depicted different variants of *dfr* and *aadA* genes with *dfrA7* at 750 bp, *dfrA1/ aadA1* at 1,600 bp and *dfrA12/ aadA2* at 2,000 bp (Table 2, Supplementary File). The amplicon length of the variable region of the integrons varied between 750 and 2,000 bp. Nucleotide sequences were compared against GenBank database by using BLAST. Sequence of variable region of class1 integron was submitted to NCBI and accession numbers were obtained (KY706079, KY706080, KY753816). Sequence analysis of the variable region of the class 1 integron have demonstrated the presence of nine different cassette combinations for seven different genes namely *dfrA7* (9), *aadA* (10), *dfrII* (13), *dfrA7*+ *aadA* (5), *dfrII*+ *aadA* (7), *dfrA7*+ *dfrA1/ aadA1* (2), *dfrA1*+*aadA1* (3), *dfrA12*+*aadA2* (10), and *dfrA 7*+*dfrA12*+*aadA2* (2). Most of the gene cassettes found within the variable region of class 1 integrons in our isolates corresponded to different variants of *dfrA* and *aadA* genes. The *dfr* gene cassette (*dfrA1, -A7, -A12, -B1, -B2, and -B3*) was found to be rampant as compared to *aadA* gene cassette. Among the integron-positive isolates, 61.8% isolates carried gene cassettes with *aadA1/2* (40.9%), *dfrII* (17.2%), and *dfrA* (31.8%).

### Detection of genes encoding resistance to beta-lactamases, tetracycline, gentamicin, and sulfonamide

Table 5 shows the distribution of antibiotic resistance genes detected in the EPEC and non-EPEC isolates among diarrheagenic cases and healthy controls. Resistance was more commonly seen in typical EPEC of diarrheagenic cases. High frequency of resistance was observed against ampicillin, sulfonamides and tetracycline. Frequency of antibiotic resistance genes detected were compared as follows (% distribution among EPEC/ % distribution among non-EPEC): *TEM* (35.6/57.5), *SHV* (31/36.3), *CTX* (19.5/18.1), *OXA* (16/18.1), *NDM-1* (12.6/3), *IMP* (11.5/9), *VIM* (17.2/12.1), *ACT* (11.5/15.1), *DHA* (4.5/6), *CMY* (4.5/18.1), *sul 1* (49.4/42.4), *tet A* (21.8/15.1) and *aacC 1* (18.3/21.2). In healthy controls representing EPEC, 6/23 (26%) isolates were positive for *NDM* and *VIM* each, *IMP* gene was seen in 5/23 (21.7%) while AmpC encoding *ACT* and *DHA* gene were positive in 4/23 (17.4%) and 1/23 (4.3%) isolates respectively. In healthy controls representing non-EPEC, only 1/17 (5.8%) isolate was positive for *VIM* and *IMP* each, 2 (11.7%) isolates were positive for ACT and DHA genes each while *CMY* gene was positive in 3/17 (17.6%) isolates. Among total EPEC (87) and total non-EPEC (33) isolates, carriage of resistance genes; *TEM* was found in 31(35.6%) and 19 (57.5%) isolates, while *CMY* was found in 4 (4.5%) and 6 (18.1%) isolates respectively, which were statistically significant (*p* < 0.05) as shown in Table 5.

**Table 5:** **Distribition of antibiotic resistance genes**.

**Genes**	**EPEC**		**Non-EPEC**		**Total EPEC ^*A*1+*B*1^ (*n* = 87)%**	**Total Non- EPEC ^*A*2+*B*2^ (*n* = 33)%**	***P*-value**
	**DC ^A1^(*n* = 64)%**	**HC ^B1^ (*n* = 23)%**	**DC ^A2^ (*n* = 16)%**	**HC ^B2^(*n* = 17)%**			
*Tem*	23 (35.9)	8 (34.7)	11 (68.7)	8 (47)	31 (35.6)	19 (57.5)	0.04^*^
*Shv*	22 (34.3)	5 (21.7)	6 (37.5)	6 (35.2)	27 (31)	12 (36.3)	0.58
*Ctx-m*	12 (18.7)	5 (21.7)	3 (18.7)	3 (17.6)	17 (19.5)	6 (18.1)	0.88
*Oxa*	12 (18.7)	2 (8.6)	1 (6.2)	5 (29.4)	14 (16)	6 (18.1)	0.77
*NDM-1*	5 (7.8)	6 (26)	1 (6.2)	0 (0)	11 (12.6)	1 (3)	0.12
*IMP*	5 (7.8)	5 (21.7)	2 (12.5)	1 (5.8)	10 (11.5)	3 (9)	0.74
*VIM*	9 (14)	6 (26)	3 (18.7)	1 (5.8)	15 (17.2)	4 (12.1)	0.51
*ACT*	6 (9.3)	4 (17.4)	3 (18.7)	2 (11.7)	10 (11.5)	5 (15.1)	0.59
*DHA*	3 (4.6)	1 (4.3)	1 (6.2)	2 (11.7)	4 (4.5)	2 (6)	0.73
*CMY*	4 (6.2)	0 (0)	3 (18.7)	3 (17.6)	4 (4.5)	6 (18.1)	0.03^*^
*sul1*	30 (46.8)	13 (56.5)	8 (50)	6 (35.2)	43 (49.4)	14 (42.4)	0.49
*tetA*	17 (26.5)	2 (8.6)	2 (12.5)	3 (17.6)	19 (21.8)	5 (15.1)	0.43
*aacC1*	15 (23.4)	1 (4.3)	6 (37.5)	1 (5.8)	16 (18.3)	7 (21.2)	0.72

*DC, diarrheagenic cases; HC, healthy controls; EPEC, enteropathogenic E. coli*.

* *Statistically significant*.

**Figure 3:**
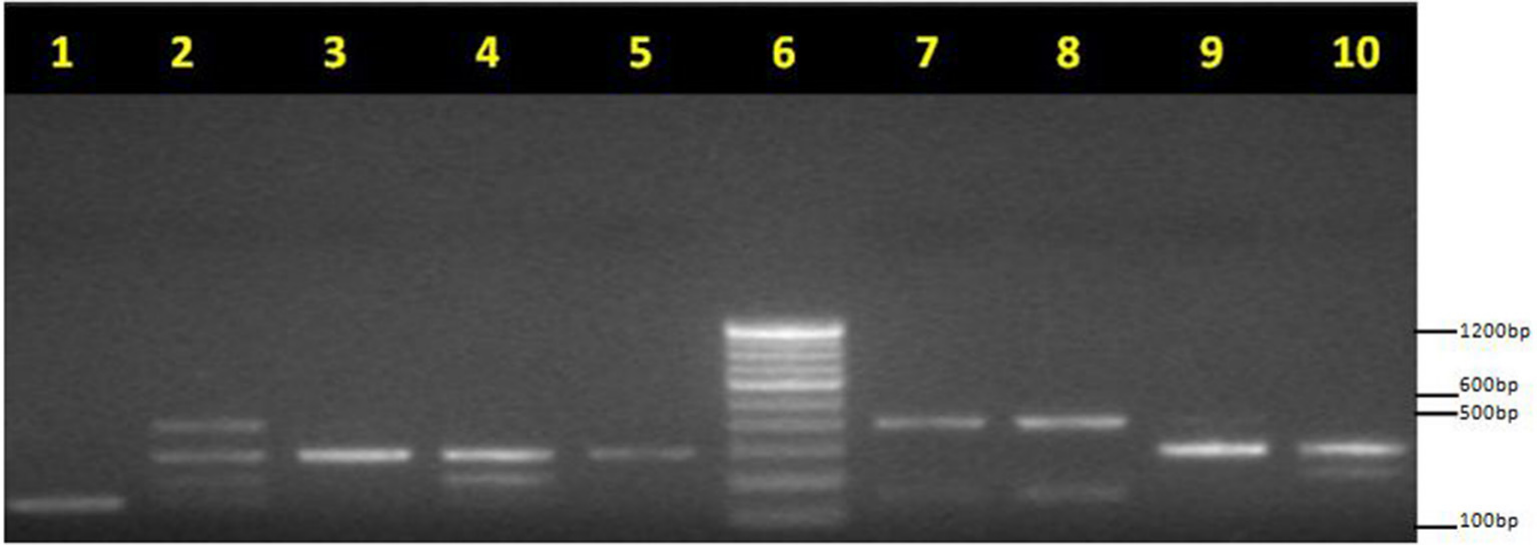
**Phylogrouping of EPEC isolates: Quadruplex PCR profiles of new Clermont phylotyping method**. *arpA* (400 bp), *chuA* (288 bp), *yjaA* (211 bp), and TspE4.C2 (152 bp) Lane 1, unknown (− − − +); lane 2, unknown (+ + + +); lane 3, group F (− + − −); lane 4, group B2 (− + + −); lane 5, group F (− + − −); lane 6, M: molecular weight marker (100 bp, Fermentas). lane 7, group B1 (+ − − +); lane 8, group B1 (+ − − +); lane 9, group F (− + − −); lane 10, group B2 (− + + −). PCR products were loaded on 1.5% agarose gel after electrophoresis; gels were photographed under UV light.

Figure 3 represents the result of quadruplex PCR, for the phylogroups under study on 1.5% agarose gel. According to Clermont et al. (2013), four of the quadruplex genotypes (− − − +, − − + +, + − + + and + + + +) for *arpA* (400 bp), *chuA* (288 bp), *yjaA* (211 bp) and *TspE4.C2* (152 bp) genes respectively have not been detected till date. In Table 6, the phylogenetic groups as determined by Clermont et al. (2013) showed the prevalence of phylogenetic group B2 in EPEC (37.9%), followed by groups B1, A, D, F, C and E in our study. The similar pattern was observed in the non-EPEC isolates with prevalence of group B2 followed by B1, F and A as shown in Table 6. All the isolates were assigned a phylogroup but eight and three of the isolates remained unclassified in EPEC and non-EPEC isolates. None of the isolates were confirmed to belong phylogroup Clade I, neither in diarrheagenic cases nor in healthy controls. However, for EPEC and non-EPEC isolates none of the phylogroups were found to be significantly associated. The absence of any significant difference between phylogroups detected in EPEC and non-EPEC isolates may be due to the small number of strains/children recruited in our study.

**Table 6:** **Distribution of phylogenetic groups in EPEC and non-EPEC isolates**.

	**EPEC**			**non-EPEC**		
**Phylogroup**	DC^*^ (*n* = 64)	HC^*^ (*n* = 23)	T^*^ (*n* = 87)	DC^*^ (*n* = 16)	HC^*^ (*n* = 17)	T^*^ (*n* = 33)
*A*	11 (17.1)	5 (21.7)	16(18.3)	1(6.2)	2(11.7)	3(9.0)
*B1*	12 (18.7)	4 (17.3)	16 (18.3)	3(18.7)	7(41.1)	10(30.3)
*B2*	25(39.0)	8(34.7)	33 (37.9)	7(43.7)	4(23.5)	11(33.3)
*C*	2 (3.1)	1 (4.3)	3 (3.4)	0(0)	0(0)	0(0)
*D*	4 (6.2)	2 (8.6)	6 (6.8)	0(0)	0(0)	0(0)
*E*	1 (1.5)	1 (4.3)	2 (2.2)	1(6.2)	1(5.8)	2(11.7)
*F*	3 (4.6)	0	3 (3.4)	2(12.5)	2(11.7)	4(23.5)
*Clade 1*	0	0	0	0(0)	0(0)	0(0)
*Unclassified*	6(9.3)	2 (8.6)	8 (9.1)	2(12.5)	1(5.8)	3(9.0)

*Gene frequencies are presented as absolute numbers with percentage in parentheses*.

* DC, diarrheagenic cases;

* HC, healthy controls;

* *T, total*.

In Table 7, frequency of antimicrobial resistance among the prevalent phylogenetic groups in EPEC and non-EPEC isolates from diarrheagenic cases and healthy controls is shown. For EPEC isolates, high prevalence of resistance was observed against cefotaxime (71.2%) followed by gentamicin (33.3%), amikacin (26.4%), ampicillin (23%), Piperacillin/tazobactam (22.9%), norfloxacin (22.9%), nalidixic acid (16%), ciprofloxacin (13.7%), ceftazidime (13.7%), aztreonam (12.6%) and imipenem (9.1%). A significant relationship was found between antibiotic resistance (cefotaxime, gentamicin, amikacin, ampicillin and Piperacillin/tazobactam) and major phylogenetic groups detected (A, B1, and B2) in EPEC isolates. For non-EPEC isolates, ampicillin showed 30.3% resistance (*P* < 0.05) followed by norfloxacin (18.1%), cefotaxime (15.1%), gentamicin (6%) and Piperacillin/tazobactam (3%) as shown in Table 7.

**Table 7:** **Frequency of antimicrobial resistance among the prevalent phylogenetic groups in EPEC and non-EPEC isolates from diarrheagenic cases and healthy controls**.

**Antibiotics**	**EPEC (*****n*** = **87)**			**non-EPEC (*****n*** = **33)**		
	**Total resistant (%)**	**Phylogroups A+B1+B2 Total (%)**	**Association of resistance with phylogroups (*P*-value)**	**Total resistant (%)**	**Phylogroups A+B1+B2 Total (%)**	**Association of resistance with phylogroups(*P*-value)**
Norfloxacin	20(22.9)	12(13.7)	0.619	6(18.1)	6(18.1)	>0.99
Cefotaxime	62(71.2)	21(24.1)	0.002^*^	5(15.1)	5(15.1)	>0.99
Imipenem	14 (16.1)	3(3.4)	0.515	4 (12.1)	0	N.A
Meropenem	3(3.4)	0	0.207	0	0	N.A
Ceftazidime	12(13.7)	2(2.2)	0.090	0	0	N.A
Azetronam	11(12.6)	2(2.2)	0.127	0	0	N.A
Nalidixic acid	14(16)	2(2.2)	0.044	0	0	N.A
Amoxicillin	3(3.4)	0	0.207	0	0	N.A
Gentamicin	29 (33.3)	7(8.0)	0.014^*^	2(6)	2(6)	>0.99
Ciprofloxacin	12(13.7)	10(11.5)	0.657	0	0	N.A
Ampicillin	20 (23)	0	0.001^*^	10 (30.3)	0	0.001^*^
Amikacin	23(26.4)	3(3.4)	0.004^*^	0	0	N.A
Polymixin B	1(1.1)	0	0.648	0	0	N.A
Cefotaxime+ clavulanic acid	1(1.1)	0	0.648	0	0	N.A
Ceftriaxone	2(2.2)	0	0.419	0	0	N.A
Piperacillin+tazobactam	20 (22.9)	3 (3.4)	0.010^*^	1(3)	1(3)	>0.99

* *significant p-value*.

*Frequencies are presented as absolute numbers with percentage in parentheses*.

## Discussion

EPEC is the most common cause of childhood diarrhea mainly in developing countries resulting in 30–40% deaths worldwide as they are prevalent in both community and hospital settings (Ochoa and Contreras, 2011; Ramana and Tamanna, 2012). Our study, focused on the EPEC isolated from stool samples of diarrheagenic and healthy children of up to 5 years of age for its virulence, distribution and characterization of integrons, their phylogenetic background, antibiogram and antibiotic resistance genes associated with them. EPEC strains are non-invasive and cause watery diarrhea after formation of attaching and effacing (A/E) lesions (Nataro and Kaper, 1998; Ochoa and Contreras, 2011). As indicated in Table 1, EPEC were found to be significantly present in diarrheagenic cases (80%) and healthy controls (57.5%) which is in accordance with other Indian studies [36.58% and 0% (Tilak and Mudaliar, 2012); 16 and 3% (Hegde et al., 2012)] and worldwide studies viz. 56.7 and 8.3% in Iran, 18.3 and 5.6% in Brazil, 3.95 and 0% in Hong Kong, 13.7 and 5.5% in Tunisia and 6.17 and 1.96% in Rondonia, Western Amazon region, Brazil in diarrheagenic cases and healthy controls respectively (Tsukamoto et al., 1992; Biswas et al., 1996; Alikhani et al., 2006; Orlandi et al., 2006; Ben Salem-Ben Nejma et al., 2014).

Typical EPEC isolates show lower occurrence in diarrheagenic cases and healthy controls with 18.75% and 2.5%, as compared to atypical EPEC with 25% and 37.5% isolates respectively, which is in accordance with previous reported studies (Afset et al., 2004; Ochoa et al., 2008; Nair et al., 2010). In our study, aEPEC was found predominantly in healthy controls which were similar to previous studies indicating their survival as colonizers in the intestinal mucosa (Dias et al., 2016). The higher rate of aEPEC in healthy controls also represents asymptomatic carriage rather than symptomatic infection that under favorable host conditions may become pathogenic (Afset et al., 2004; Nair et al., 2010). Since, atypical EPEC strains have not been confirmed to be nonpathogenic, or less pathogenic as compared to typical ones the only possibility is that they have adopted other virulence factors for the loss of *EAF* plasmid (Scaletsky et al., 2010). EPEC strains are classified into typical and atypical based on the presence of the large virulence EPEC adherence factor (*EAF*) plasmid (Nataro and Kaper, 1998). The EAF plasmid encodes a type IV fimbria known as the bundle-forming pilus (*bfp*) that mediate atypical EPEC to cause persistent diarrhea although the mechanism by which it establishes infection, is still unclear (Giron et al., 1991; Donnenberg et al., 1992; Bieber et al., 1998; Knutton et al., 1999; Afset et al., 2004).

Hall and Collis (1995) showed class 1 and 2 integrons to be prevalent in enteropathogens. Among EPEC isolates, class 1 integron was significantly associated (Table 2). Class 3 integron was not found in any isolate as reported by other studies (White et al., 2001; Yu et al., 2003; Machado et al., 2005; Skurnik et al., 2005; Su et al., 2006; Vinue et al., 2008; Muhammad et al., 2011; Rezaee et al., 2011). Contradicting to the observation, a single study in Iran showed prevalence of class 3 integron with 26.09% (Kargar et al., 2014). Our study also indicated that integrons exist in *E. coli* isolated from healthy controls as well. Frequency of class 1 integron in healthy controls was comparable (37.5%) to other studies with the highest prevalence of 85.5% reported from China (Su et al., 2006), followed by 49% from Estonia (Sepp et al., 2009), 29% from Spain (Vinue et al., 2008) and 21% from India (Dureja et al., 2014). Herein, after comparing the presence of integrons in cases and controls, the occurrence of class 1 integrons in healthy controls suggests a possible acquisition of resistance genes circulating in the hospital environment and may act as potential reservoir of integrons in maintaining a constant horizontal exchange of these genes (Roe et al., 2003). The integrons highlight the emergence of multidrug resistance, as usage of one antibiotic may activate the expression and transfer of a whole gene cassette emphasizing the association of class 1 integrons to MDR (Leverstein-van Hall et al., 2003; Norrby, 2005).

We cannot deny the fact that EPEC isolates become predominant under the antibiotic influence, and it facilitates their selective proliferation and colonization in gastrointestinal tracts of children. Such resistance with slow reversal rate has higher chances of persistence in the bacterial population (Andersson and Hughes, 2010). Higher resistance to some antimicrobial agents (gentamicin, cefotaxime, ceftazidime, nalidixic acid, and aztreonam) among integron-positive isolates (91.6%) indicated the involvement of resistance genes in the same mobile elements that carry integrons as shown in Table 3. Suspected localization of resistance genes in the conserved or variable region of integrons or the involvement of resistance genes in the same mobile elements that carry integrons may confer higher resistance. Among EPEC isolates, (40/87) 45.97 percent isolates, showed MDR phenotype with higher antibiotic resistance to cefotaxime followed by gentamicin, ampicillin, norfloxacin, amikacin, Piperacillin+tazobactam, imipenem, nalidixic acid, ciprofloxacin, as reported by others (Saravanan and Raveendaran, 2013; Lin et al., 2014; Wang et al., 2015). Such high cefotaxime-resistant (CTX-R) population of enterobacteriaceae, constitute a reservoir for transmission that may remain unidentified in hospitals which do not implement active surveillance testing (Tarchouna et al., 2014). In our study, less than half of the isolates were multidrug resistant (45.83%) which has also been reported by other workers (Elsharkawy et al., 2013). Class 1 integron was predominant among EPEC isolates, and significantly associated with MDR (*P* < 0.05) as shown in Table 4. There are studies which also reported association between MDR and integrons (Bakhshi et al., 2014; Kargar et al., 2014; Malek et al., 2015). Occurrence of gene cassettes in our study was different in terms of number and gene combinations as reported by many other researchers (White et al., 2001). The *dfr* gene cassette (*dfrA1, -A7, -A12*, -B1, -B2, and -B3) was found to be prevalent as compared to the *aadA* gene cassette, (Table 2, Supplementary File) which is in agreement with Malek et al. (2015).

Acquisition of tetracycline, sulphonamide and gentamicin resistance genes are also of great concern (Table 5). Tetracycline resistance gene *tet(A)* and class 1 integrons usually share the same conjugative plasmid (Sunde and Norstrom, 2006), while the acquisition of gentamicin resistance genes are yet to be established. The prevalence of *sul1* gene in our integron-positive healthy isolates was higher by 47.5% to previously reported study (Infante et al., 2005). High prevalence of sulphonamide resistance in healthy controls suggests that commensal strains could also represent an important reservoir of these resistance determinants. A significant association between resistance to aminoglycosides tested (gentamicin) and the presence of integron was also explainable because many aminoglycoside resistance genes have been reported within integron structures, including *aadA* and *aacA1* (Martinez-Freijo et al., 1999).

*Escherichia coli* have the potential to spread outside the hospital environment also as described by Banerjee and Johnson (2014). In our study (Table 5), production of enzymes encoding beta-lactamases genes in healthy children suggests that children may be silently harboring MDR strains with prolonged colonization, which means that they could serve as potential reservoirs for these strains in the community (Zerr et al., 2014; Yaffee et al., 2016). *NDM* gene is located on transmissible plasmid along with other antibiotic resistance genes, which make their spreading easy (Kumarasamy et al., 2010). The *VIM* encoding integron structure most likely has been acquired during hospital stay and then colonized in patients before leaving (Riccio et al., 2001). The emergence of the *CMY* gene has been reported among diverse genera of the *Enterobacteriaceae* (Qin et al., 2008). The observed increase in the proportion of beta-lactamase producers among healthy controls is probably multi-factorial, resulting from the clonal spread of virulent strains, dissemination of conjugative plasmids as well as increased selection pressure (Baudry et al., 2009). There are many other factors for their dissemination like overcrowding, availability of antibiotics, low level of hygiene and weak hospital antibiotic policies (Nordmann et al., 2011).

The phylogenetic analyses as described in Table 6 showed majority of EPEC isolates belonging to one of four major phylogenetic groups (A, B1, B2, or D) in diarrheagenic cases as well as in healthy controls. In diarrheagenic cases, EPEC isolates depicted a strong association with phylogroup B2 which is in accordance with other previously reported study, followed by B1, A, D, F, C, and E (Chakraborty et al., 2015). Six (diarrheagenic cases) and two (healthy control) isolates remained unclassified, which could either be due to their rarity of occurrence or a high frequency of loss of gene leading to low existence of these phylogroups (Touchon et al., 2009). In EPEC isolates from healthy controls, B2 (34.7%) remained the predominant phylogroup, similar to other previously reported studies followed by A, B1, D, C, or E, whereas Group F was not observed in any isolate (Bailey et al., 2010; Wang et al., 2013; Rana et al., 2016). Previous studies have indicated that phylogroups B2 and D possess more virulence properties, compared to other phylogroups. Similar pattern of occurrence of phylogenetic groups in both diarrheagenic cases and healthy controls is suggestive of a homogeneous group existing in the pediatric population with an onward circulation and transfer of the similar set of virulence genes (Nguyen et al., 2010).

Table 7 shows the antibiotic resistance of all the phylogroups revealed that group B2 displayed maximum number of antibiotic resistance genes with nearly similar number of isolates in tEPEC and aEPEC category while the isolates in phylogroups A and B1 were mainly found to be linked with aEPEC (Wang et al., 2013). We found cefotaxime resistance to be significantly associated with group B2 compared to others (Soto et al., 2007; Jakobsen et al., 2010). Many virulence genes after implanting themselves into pathogenicity islands move along with integrons into the environment via horizontal gene transfer. The preponderance of strains in different phylogroups may vary due to the intensity of antibiotic resistant strains, virulence of the strains, geographical site and the site of infection (Duriez et al., 2001; Bukh et al., 2009).

A strong association between the presence of integrons and different phylogroups was found in our study (*p* = 0.004) which is also in agreement with a previous study (Mabbett et al., 2009). For EPEC isolates, we found a strong association between class 1 integron with phylogenetic group A, B1 and B2 (*P* < 0.05) while for non-EPEC isolates class 1 integron was significantly associated with only phylogenetic group A. Phylogenetic group B2 was significantly associated with class 2 integron and when both the integrons are present together for EPEC isolates (*P* < 0.05).

Essentially, our study conveys a strong link between diarrheagenic *E. coli* populations and establishment of virulence and antibiotic resistance. The epidemiological groups' existance in children determines the circumstances of persistence of the pathogen in the community. Although, quadruplex PCR has limitations of not detecting all clades, it can still be utilized to demonstrate the role of integrons in multidrug resistance within diarrheagenic and commensal *E. coli* strains. Since, integrons are common among MDR isolates, hence; they can be used as a marker for the identification of MDR isolates. Isolates carrying integrons representing multidrug resistance may be explored as a useful therapeutic target for future research using specific silencing strategies and combination antimicrobial therapy. Moreover, occurrence of resistance in healthy controls indicates that antimicrobial resistant isolates in the gut are widely circulating in the community and hence continued surveillance of antibiotic resistance will provide crucial information in developing locally appropriate guidelines for efficacious treatment of diarrheagenic *E. coli* in developing countries like India.

## Author contributions

TS and SD conceived and designed the study, analyzed/interpreted results. TS and AS collected the samples. TS and SW collected the data. AS, TS, and SW performed experiments and analyzed the data. TS, SW, and DS carried out the literature search. TS and SW gave technical support and conceptual advice. VR, SD, and TS wrote the manuscript. VR, KM, and DS participated in design and supervision of the study and revised the final version of the manuscript. KM performed statistical analysis also. AS and KM performed the manuscript editing. SD and TS supervised the study and revised the final version of the manuscript. All authors read and approved the final manuscript.

## Funding

This work was supported by Council of Scientific and Industrial Research (CSIR) Library Avenue, Pusa Road, New Delhi 110012. Project 08/532 (0007)/2011-EMR-I.

### Conflict of interest statement

The authors declare that the research was conducted in the absence of any commercial or financial relationships that could be construed as a potential conflict of interest.
