# Supplementary material for: Distribution of Integrons and Phylogenetic Groups among Enteropathogenic Escherichia coli Isolates from Children <5 Years of Age in Delhi, India
Source: Front Microbiol. 2017 Apr 10;8:561. doi: 10.3389/fmicb.2017.00561 (PMC5385330; doi:10.3389/fmicb.2017.00561)
Supplement: Supplementary file 1 [file Table1.PDF]

Table 1: Details of primers for EPEC, integrons, phylogroups, antibiotic resistance genes and gene cassettes used in this study.

Multiplex PCR	Gene	Primer sequence (5'-3')	PCR product (bp)	Annealing temperature	Reference
(SET I) EPEC	*eae*	AAACAGGTGAAACTGTTGCC<br>CTCTGCAGATTAACCCTCTGC	482	53^0C	Yu and Kaper, 1992
	Eaf	CAGGGTAAAAGAAGATGATAA<br>TATGGGGACCATGTATTATCA	397		Franke et al., 1994
	bfpA	AATGGTGCTTGCGTTGCTGC<br>GCCGCTTTATCCAACCTGGTA	326		Gunzburg et al., 1995
(SET II) INTEGRONS	Class 1 integrase gene	CAGTGGACATAAGCCTGTTC<br>CCCGAGGCATAGACTGTA	160	60^0C	Levesque et al., 1995
	Class 2 integrase gene	CAACGGAGTCATGCAGATG<br>CATTTGTGTTGTGGACGGC	403		Li et al., 2014
	Class 3 integrase gene	AGTGGGTGG CGAATGAGTG<br>TGTTCTTGTATCGGC AGGTG	600		Machado et al., 2005
(SET III) PHYLOGENETIC GROUPS (Quadruplex)	chuA.1b<br>chuA.2	ATGGTACCGGACGAACCAAC<br>TGCCGCCAGTACCAAAGACA	288	60^0C	Clermont et al., 2013
	yjaA.1b<br>yjaA.2b	CAAACGTGAAGTGTCAGGAG<br>AATGCGTTCCTCAACCTGTG	211		Clermont et al., 2013
	TspE4C2.1b<br>TspE4C2.2b	CACTATTCGTAAGGTCATCC<br>AGTTTATCGCTGCGGGTCGC	152		Clermont et al., 2013
	AceK.f<br>ArpA1.r	AACGCTATTCGCCAGCTTGC<br>TCTCCCCATACCGTACGCTA	400		Clermont et al., 2013
Group E (*arpA*)	ArpAgpE.f<br>ArpAgpE.r	GATTCCATCTTGTCAAAATATGCC<br>GAAAAGAAAAAGAATTCCCAAGAG	301		Lescat et al., 2013
Group C (*trpA*)	trpAgpC.1<br>trpAgpC. 2	AGTTTTATGCCCAGTGCGAG<br>TCTGCGCCGGTCACGCCC	219		Clermont et al., 2008
Internal control (*trpA*)	trpBA.f<br>trpBA.r	CGGCGATAAAGACATCTTCAC<br>GCAACGCGGCCTGGCGGAAG	489		Clermont et al., 2008
	**Antibiotic Resistance genes**				
**Beta-lactams** ESBL	TEM	AGTGCTGCCATAACCATGAGG<br>CTGACTCCCCGTCGTGTAGATA	431		Kim et al., 2009
	SHV	GATGAACGCTTTCCCATGATG<br>CGCTGTTATCGCTCATGGTAA	214		Kim et al., 2009
	OXA	ATTATCTACAGCAGCGCCAGTG<br>TGCATCCACGTCTTTGGTG	296		Kim et al., 2009
	CTX-M	GACAAAGAGAGTGCAACGGATG<br>TCAGTGCGATCCAGACGAAA	501		Kim et al., 2009
MBL	blaNDM-1	ATTAGCCGCTGCATTGAT<br>CATGTCGAGATAGGAAGTG	154	55^0C	Naas et al., 2011
	blaIMP	TTGACACTCCATTTACAG<br>GATTGAGAATTAAGCCACTCT	139		Dallenne et al., 2010
	blaVIM	GATGGTGTTTGGTCGCATA<br>CGAATGCGCAGCACCAG	390		Dallenne et al., 2010
AmpC	bla*CMY*	GCTGCTCAAGGAGCACAGGAT<br>CACATTGACATAGGTGTGGTGC	520	60^0C	Manoharan et al., 2012
	DHA	AACTTTCACAGGTGTGCTGGGT<br>CCGTACGCATACTGGCTTTGC	405		Manoharan et al., 2012
	ACT-1	TCGGTAAAG CCGATGTTG CGG<br>CTT CCA CTG CGG CTG CCA GTT	302		Manoharan et al., 2012
Tetracycline	TetA-F<br>TetA-R	GTAATTCTGAGCACTGTCGC<br>CTGCCTGGACAACATTGCTT	937	62	Guardabassi et al., 2000
Sulphonamides	sul1-F<br>sul2-R	TGGTGACGGTGTTCGGCATTC<br>GCGAGGGTTTCCGAGAAGGTG	789	63	Mazel et al., 2000
Gentamicin	AacC1-F<br>AacC1-R	ACCTACTCCCAACATCAGCC<br>ATATAGATCTCACTACGCGC	169	60	Van de Klundert et al., 1993
	**Gene cassettes**				
Dihydrofolate reductase	dfrII (dfrB1, dfrB2, dfrB3)	GATCACGTGCGCAAGAAATC<br>AAGCGCAGCCACAGGATAAAT	141	60^0C	Navia et al., 2003
Aminoglycoside adenylyltransferases	aadA (*aadA1* or *aadA2)*	GCTCTTCAGCAATATCACGG<br>GCAGCGCAATGACATTCTTG	282	60^0C	Hollingshead and Vapnek, 1985
(Putative protein) binds to 5' and 3' conserved sequence of class 1 integrons	hep58<br>hep59	CSGGCATCCAAGCAGCAAG<br>CSAAGCAGACTTGACCTGA	Variable (750-2000)	58^0C	Machado et al., 2005

EPEC=Enteropathogenic *E. coli,* ESBL=extended spectrum beta lactamases, MBL=metallo beta-latamases, AmpC= AmpC β-Lactamases

Table 2: Phenotypes of antibiotic resistance, presence of EPEC/non-EPEC, type of integrons, antibiotic resistance genes and gene cassettes isolated from diarrheagenic cases and healthy controls isolates of this study.

Isolates	Resistance phenotype to antibiotics*	Detection o EPEC virulence	Integron detected	Antibiotic resistance genes detected (Beta-lactamase genes and Other resistance genes)	Gene cassette inside the variable region (dfrII, *aadA*)
**Diarrheagenic cases**					
**DC 1**	NF-CTX-NA-CIP-AMK-AMP[#]	**EPEC**	**1**	*TEM, SHV, sul1*	*dfrA7*[b]
**DC 2**	CTX- AMP	**EPEC**	**1**	*ACT, DHA, TEM, tetA,*	*aadA*[a]
**DC 3**	CTX- AMP	**EPEC**	**1**	*TEM, tetA*	*aadA*[a] + *dfrA7*[b]
**DC 4**	CTX-NA-TZP[#]	**EPEC**	**1**	*SHV, CTX, sul1*	*dfrII*[a] + *aadA*[a]
**DC 5**	IPM-MEM-AZT-GEN-AMP-AMK[#]	**EPEC**	**1**	*ACT, CMY, TEM, OXA, aacC1*	*aadA*[a] +*dfrA7*[b]
**DC 6**	NF-CAZ-NA[#]	**EPEC**	**2**	*CTX, sul1*	*dfrII*[a]
**DC 7**	NF-CTX-NA-GEN-CIP[#]	**EPEC**	**1, 2**	*VIM, ACT, sul1, aacC1*	*dfrII*[a] + *aadA*[a]
**DC 8**	CTX-CIP-PB-TZP-AMP[#]	**non-EPEC**	**2**	*TEM, sul1*	ND
**DC 9**	CTX-CAZ-GEN-NA-AMK- AMP[#]	**EPEC**	**1**	*SHV, OXA, sul1, aacC1*	*dfrII*[a] + *aadA*[a]
**DC 10**	GEN-TZP	**EPEC**	**1**	*SHV, aacC1,*	*dfrA7*[b] + *dfrA1/ aadA1*[b]
**DC 11**	AZT	**EPEC**	**2**	*OXA*	ND
**DC 12**	CTX-IPM-NA-AMK[#]	**EPEC**	**1**	*sul1*	*dfrA1/ aadA1*[b]
**DC 13**	CTX-CAZ-GEN-NA-AMK[#]	**non-EPEC**	**1**	*CMY, sul1, aacC1*	ND
**DC 14**	CTX-IPM-MEM-CAZ-TZP[#]	**EPEC**	**1**	*tetA*	*dfrA7*[b] +*dfrA12/ aadA2*[b]
**DC 15**	NF-NA-TZP-AMP[#]	**EPEC**	**1, 2**	*CMY, TEM, SHV, CTX, sul1,*	*aadA*[a]
**DC 16**	CTX-NA-GEN-CIP-TZP- AMP[#]	**non-EPEC**	**2**	*TEM, SHV, tetA, sul1, aacC1*	ND
**DC 17**	CTX-CIP-AMK[#]	**EPEC**	**1**	*ACT, CMY, sul1*	ND
**DC 18**	CIP	**EPEC**	**1, 2**	*tetA,sul1*	*dfrII*[a] + *dfrA7*[b]
**DC 19**	AZT- AMP	**EPEC**	**2**	*DHA, CMY, TEM, OXA, tetA*	ND
**DC 20**	GEN-NA	**EPEC**	**1**	*TEM, sul1, aacC1*	ND
**DC 21**	NF-CTX-NA-AMP-TZP-AMP[#]	**non-EPEC**	**1, 2**	*TEM, SHV, sul1,*	*aadA*[a]
**DC 22**	CTX-CAZ-AMK[#]	**EPEC**	**1**	*TEM, CTX, tetA*	*dfrII*[a] + *aadA*[a]
**DC 23**	CTX	**EPEC**	**1**	*CTX, tetA,*	*dfrA1/ aadA1*[b]
**DC 24**	NF-CTX-IPM- AMP[#]	**non-EPEC**	**1, 2**	*ACT, TEM, SHV, OXA, sul1*	*aadA*[a]
**DC 25**	CTX-AMC-GEN-AMK-TZP-AMP-IPM[#]	**EPEC**	**1**	*VIM, TEM, SHV, tetA, aacC1*	*dfrA12/ aadA2*[b]
**DC 26**	CTX	**EPEC**	**2**	*ACT, DHA, SHV*	ND
**DC 27**	NF-CTX-CAZ-AZT-NA[#]	**EPEC**	**1**	*IMP, VIM, sul1*	ND
**DC 28**	CIP-AMK	**EPEC**	**1, 2**	*SHV, tetA*	*dfrII*[a] + *aadA*[a]
**DC 29**	CTX-GEN	**EPEC**	**1**	*TEM, aacC1*	*dfrA1/ aadA1*[b]
**DC 30**	GEN-AMP	**EPEC**	**1**	*TEM, SHV, CTX, tetA, aacC1*	ND

**DC 31**	NA-TZP	**non-EPEC**	**1**	*TEM, CTX, sul1,*	*dfrA7* [b]
**DC 32**	NF-CTX-GEN#	**EPEC**	**1**	*sul1, aacC1*	*dfrA12/ aadA2* [b]
**DC 33**	CTX-IPM-CAZ#	**non-EPEC**	**2**	*IMP, DHA, CMY, TEM*	ND
**DC 34**	CTX-NA	**EPEC**	**1, 2**	*NDM, VIM, sul1*	*dfrII* [a]
**DC 35**	CTX	**EPEC**	**1**	*tetA*	ND
**DC 36**	GEN-TZP	**non-EPEC**	**1**	*ACT*	*dfrA1/ aadA1* [b]
**DC 37**	NF-NA	**EPEC**	**1**	*sul1*	ND
**DC 38**	CTX-CIP	**EPEC**	**2**	*TEM, SHV, tetA, sul1*	ND
**DC 39**	CTX-CAZ-NA-GEN#	**EPEC**	**1, 2**	*aacC1*	dfrII [a]
**DC 40**	CTX-AZT-NA-GEN-CIP#	**EPEC**	**1**	*TEM, OXA, sul1,*	*dfrA12/ aadA2* [b]
**DC 41**	CTX-AZT-NA-TZP#	**non-EPEC**	**1**	*TEM, ACT, CMY, tetA, sul1*	ND
**DC 42**	CTX-AMK	**EPEC**	**2**	*TEM*	ND
**DC 43**	CIP- AMP- IPM#	**non-EPEC**	**1**	*IMP, VIM, TEM, SHV, sul1,*	*aadA* [a] + *dfrA7* [b]
**DC 44**	GEN	**EPEC**	**1**	*aacC1*	*dfrII* [a]
**DC 45**	NF-CTX	**EPEC**	**1, 2**	*SHV, sul1*	ND
**DC 46**	CTX-GEN-AMK-TZP#	**non-EPEC**	**1**	*TEM, CTX, aacC1*	*dfrII* [a]
**DC 47**	CTX-IPM-NA-AMK-CCA#	**EPEC**	**1**	*VIM, SHV, OXA, tetA, sul1*	ND
**DC 48**	CTX-GEN-IPM#	**EPEC**	**1**	*NDM, VIM, SHV, OXA, aacC1*	ND
**DC 49**	CTX-AZT-GEN-TZP#	**non-EPEC**	**1**	*aacC1, CMY*	*aadA* [a]
**DC 50**	AZT-NA-AMP#	**EPEC**	**1**	*TEM, CTX, sul1,*	*dfrA1/ aadA1* [b]
**DC 51**	CTX-AMK	**EPEC**	**1**	*NDM, IMP, ACT, tetA,*	*dfrA7* [b]
**DC 52**	NF-CAZ-IPM#	**EPEC**	**1**	*IMP, VIM, TEM, sul1*	*dfrII* [a] + *aadA* [a]
**DC 53**	CTX-GEN-CRO#	**non-EPEC**	**1, 2**	*IMP, VIM, aacC1*	*aadA* [a]
**DC 54**	NF-CTX-GEN-NA-TZP#	**EPEC**	**1, 2**	*ACT, CMY, SHV, sul1, aacC1*	ND
**DC 55**	IPM-AMC-AMK-TZP- AMP #	**EPEC**	**1, 2**	*TEM, SHV, tetA*	ND
**DC 56**	CTX-IPM-MEM-CAZ#	**EPEC**	**1**	*NDM, VIM*	*aadA* [a] + *dfrA7* [b]
**DC 57**	CTX-AMK	**non-EPEC**	**1**	*VIM*	ND
**DC 58**	CTX-NA-AMK#	**EPEC**	**1**	*CTX, OXA, tetA,*	*dfrA12/ aadA2* [b]
**DC 59**	CTX -AMP	**non-EPEC**	**2**	*TEM, SHV*	ND
**DC 60**	**CTX**-GEN-AMP#	**non-EPEC**	**1**	*NDM, TEM, SHV, CTX, aacC1*	ND
**DC 61**	CTX-AMP-IPM#	**EPEC**	**2**	*NDM, IMP, CTX, OXA, tetA*	ND
**DC 62**	CTX-NA-TZP#	**EPEC**	**1**	*sul1,*	*dfrII* [a]
**DC 63**	NF-CTX-AZT-TZP#	**EPEC**	**1**	*SHV, CTX, sul1,*	*dfrA7* [b]
**DC 64**	CTX-GEN	**EPEC**	**2**	*aacC1,*	*dfrA12/ aadA2* [b]
**DC 65**	CTX-TZP	**EPEC**	**1**	*ACT, CMY, tetA*	ND
**DC 66**	NF-CTX-NA-AMK#	**EPEC**	**2**	*sul1*	ND
**DC 67**	NF-CTX-AMP#	**EPEC**	**1**	*tetA, sul1*	*dfrA7* [b]
**DC 68**	CTX-AMK-TZP-AMP #	**EPEC**	**1**	*TEM, SHV*	*dfrII* [a] + *aadA* [a]
**DC 69**	CAZ-GEN-NA-AMP #	**EPEC**	**1**	*ACT, TEM, SHV, CTX, sul1, aacC1*	*dfrII* [a]

**DC 70**	CTX-NA	**EPEC**	**1**	*None*	*dfrA1/ aadA1* [b]
**DC 71**	CTX-AMK-AMP[#]	**EPEC**	**1**	*TEM, SHV, CTX, tetA,*	*aadA*
**DC 72**	CTX-GEN-NA[#]	**EPEC**	**1**	*TEM, OXA, sul1*	*dfrA7* [b]
**DC 73**	CTX-AZT-GEN-NA[#]	**EPEC**	**2**	*VIM, SHV*	ND
**DC 74**	CTX-GEN-NA[#]	**EPEC**	**1**	*SHV, CTX, sul1, aacC1*	*dfrA7* [b]
**DC 75**	CTX-AMK	**EPEC**	**2**	*DHA, TEM, tetA*	ND
**DC 76**	CTX-NA	**EPEC**	**1**	*ACT, sul1*	*dfrA12/ aadA2* [b]
**DC 77**	CTX	**EPEC**	**1**	*CMY, CTX, OXA*	*dfrA7* [b] *+ dfrA1/ aadA1* [b]
**DC 78**	CTX-AMC-GEN-NA-AMK[#]	**EPEC**	**1**	*DHA, sul1, aacC1*	ND
**DC 79**	CTX-GEN-NA- IPM [#]	**EPEC**	**1**	*IMP, VIM, TEM, OXA, sul1, aacC1*	*dfrA1/ aadA1* [b]
**DC 80**	NF-CAZ-NA-AMK[#]	**EPEC**	**2**	*CTX, sul1*	ND
**Healthy controls**					
**HC 1**	NF-GEN-NA[#]	**EPEC**	**1**	*sul1, aacC1*	ND
**HC 2**	NONE	**non-EPEC**	**1**	*ACT, SHV*	ND
**HC 3**	CTX-NA-AMP[#]	**non-EPEC**	**1, 2**	*TEM, SHV, tetA, sul1*	ND
**HC 4**	NA	**non-EPEC**	**1**	*TEM*	*dfrA7* [b]
**HC 5**	NA	**non-EPEC**	**None**	*SHV, CTX, OXA*	ND
**HC 6**	NA-TZP	**non-EPEC**	**None**	*NDM, ACT, CTX, sul1*	ND
**HC 7**	NF-GEN- AMP- IPM[#]	**non-EPEC**	**2**	*IMP, VIM, ACT, CMY, TEM, SHV, OXA, sul1, aacC1*	*dfrII* [a]
**HC 8**	NF	**EPEC**	**2**	*NDM, sul1*	ND
**HC 9**	NF-CIP	**EPEC**	**1**	*SHV, sul1*	dfrA7 [b]
**HC 10**	NONE	**EPEC**	**1**	*CTX*	*dfrA1/ aadA1* [b]
**HC 11**	NA	**EPEC**	**2**	*DHA, sul 1*	ND
**HC 12**	CTX-NA-CRO-AMP [#]	**non-EPEC**	**None**	*TEM, SHV, OXA, tetA, sul1*	ND
**HC 13**	CTX-NA	**non-EPEC**	**2**	*CTX*	*dfrII* [a]
**HC 14**	NONE	**non-EPEC**	**1**	*SHV, OXA*	*aadA* [a]
**HC 15**	NONE	**EPEC**	**1**	*VIM, CMY, CTX*	*dfrII+aadA* [a]
**HC 16**	NA- IPM	**EPEC**	**2**	*NDM, VIM, TEM, OXA,sul1*	*dfrII* [a]
**HC 17**	NONE	**EPEC**	**1**	*IMP*	*dfrA7* [b] *+ dfrA12/ aadA2* [b]
**HC 18**	NA	**EPEC**	**None**	*TEM*	ND
**HC 19**	NA	**EPEC**	**None**	*sul1*	*aadA* [a]
**HC 20**	NONE	**non-EPEC**	**1**	*ACT, CMY*	ND
**HC 21**	CTX	**non-EPEC**	**None**	*TEM, tetA*	ND
**HC 22**	NA	**EPEC**	**1**	*SHV, sul1*	*dfrA12/ aadA2* [b]
**HC 23**	NF- AMP	**EPEC**	**1**	*ACT, DHA, TEM, SHV, sul1*	*aadA* [a]
**HC 24**	NONE	**EPEC**	**2**	*NDM, VIM, ACT*	ND
**HC 25**	NA-TZP	**EPEC**	**None**	*IMP, VIM, sul1*	ND
**HC 26**	CTX-NA-IPM[#]	**EPEC**	**1, 2**	*NDM, VIM, DHA, CMY, CTX, sul1*	ND
**HC 27**	NF- AMP	**EPEC**	**2**	*TEM, SHV, sul1*	*dfrII* [a]

**HC 28**	NONE	**non-EPEC**	**None**	*None*	ND
**HC 29**	NF	**non-EPEC**	**None**	*ACT, DHA, sul1*	ND
**HC 30**	NA	**non-EPEC**	**1, 2**	*TEM, sul1*	*dfrA12/ aadA2* [b]
**HC 31**	NONE	**non-EPEC**	**2**	*TEM*	*dfrII* [a]
**HC 32**	CTX- AMP	**EPEC**	**None**	*TEM, SHV, tetA*	ND
**HC 33**	NF-CTX	**EPEC**	**2**	*IMP, VIM, tetA, sul1*	ND
**HC 34**	IPM	**EPEC**	**1, 2**	*NDM, IMP, ACT*	ND
**HC 35**	NA	**EPEC**	**2**	*IMP, sul1*	*dfrII* [a]
**HC 36**	NONE	**non-EPEC**	**1**	*TEM, OXA*	*aadA* [a]
**HC 37**	NA	**EPEC**	**1, 2**	*CTX*	*dfrA12/ aadA2* [b]
**HC 38**	NA	**non-EPEC**	**1**	*DHA, CMY*	*dfrA12/ aadA2* [b]
**HC 39**	NF- AMP	**EPEC**	**1**	*TEM, SHV*	*dfrA1/ aadA1* [b]
**HC 40**	NF	**EPEC**	**1**	*TEM, CTX, OXA, sul1*	ND

DC= diarrheagenic cases
HC=healthy controls
**AMK* amikacin, *AMP* ampicillin, AZT azetronam, *CAZ* ceftazidime, *CIP* ciprofloxacin, *GEN* gentamicin, *IPM* imipenem, *MEM* meropenem, *TZP* piperacillin+tazobactam, CCA Cefotaxime + clavulanic acid, NA Nalidixic acid, AZT Azetronam, PB Polymixin B, CTX Cefotaxime, NF Norfloxacin, CRO Ceftriaxone,
# Multidrug resistant strains
a= this gene cassette was determined by PCR using specific primers for *dfrII (dfrB1, dfrB2, dfrB3*) and *aadA* (*aadA1* or *aadA2)* gene but it was not confirmed by sequencing.
b= this gene cassette was determined by PCR using specific primers for *hep58* and *hep 59* which binds to conserved sequence of 5' and 3' region of integron 1 and sequencing determined it. Variable region (750-2000) size was estimated by agarose gel electrophoresis.
ND=Not Determined.

Figure 1: Uniplex PCR for EPEC virulence genes on 1.5 % agarose gel. Amplified products of EPEC virulence genes; lane 1; molecular weight marker (100 bp, Fermentas), lane 2; *eaf* (397bp), lane 3; *bfpA* (326bp) and lane 4; *eae* (482 bp).

Figure 2: **Uniplex PCR for integrase genes**. Agarose gel electrophoresis of integrase gene polymerase chain reaction (PCR) amplification products on 1.5 % agarose gel. Uniplex PCR products of class 1 integrase of 170 bp (lanes 1-8): lane 9, molecular weight marker (100 bp, Fermentas) and class 2 integrase genes of 403 bp (lane 10-17).
