# Supplementary figures and images for: Distribution of Integrons and Phylogenetic Groups among Enteropathogenic Escherichia coli Isolates from Children <5 Years of Age in Delhi, India
Source: Front Microbiol. 2017 Apr 10;8:561. doi: 10.3389/fmicb.2017.00561 (PMC5385330; doi:10.3389/fmicb.2017.00561)

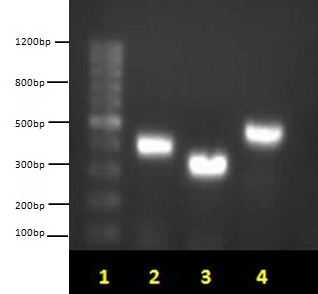

Supplement: Supplementary file 2 [file Image1.JPEG]

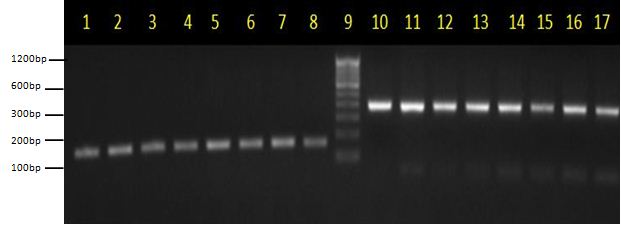

Supplement: Supplementary file 3 [file Image2.JPEG]
